# Psychological and biological mechanisms linking trauma with cardiovascular disease risk

**DOI:** 10.1038/s41398-023-02330-8

**Published:** 2023-01-27

**Authors:** Jennifer A. Sumner, Shiloh Cleveland, Tiffany Chen, Jaimie L. Gradus

**Affiliations:** 1 Department of Psychology, University of California, Los Angeles, Los Angeles, CA USA; 2 Department of Epidemiology, Boston University School of Public Health, Boston, MA USA

**Keywords:** Diseases, Psychology

## Abstract

Cardiovascular disease (CVD) is the leading cause of death and disability worldwide, and experiences of psychological trauma have been associated with subsequent CVD onset. Identifying key pathways connecting trauma with CVD has the potential to inform more targeted screening and intervention efforts to offset elevated cardiovascular risk. In this narrative review, we summarize the evidence for key psychological and biological mechanisms linking experiences of trauma with CVD risk. Additionally, we describe various methodologies for measuring these mechanisms in an effort to inform future research related to potential pathways. With regard to mechanisms involving posttraumatic psychopathology, the vast majority of research on psychological distress after trauma and CVD has focused on posttraumatic stress disorder (PTSD), even though posttraumatic psychopathology can manifest in other ways as well. Substantial evidence suggests that PTSD predicts the onset of a range of cardiovascular outcomes in trauma-exposed men and women, yet more research is needed to better understand posttraumatic psychopathology more comprehensively and how it may relate to CVD. Further, dysregulation of numerous biological systems may occur after trauma and in the presence of posttraumatic psychopathology; these processes of immune system dysregulation and elevated inflammation, oxidative stress, mitochondrial dysfunction, renin-angiotensin system dysregulation, and accelerated biological aging may all contribute to subsequent cardiovascular risk, although more research on these pathways in the context of traumatic stress is needed. Given that many of these mechanisms are closely intertwined, future research using a systems biology approach may prove fruitful for elucidating how processes unfold to contribute to CVD after trauma.

## Introduction

Despite advances in prevention and intervention, cardiovascular disease (CVD), encompassing a range of disorders of the heart and blood vessels, remains the leading cause of death and disability worldwide [1, 2]. Globally, CVD accounts for approximately one-third of all deaths; in the United States, CVD claims more lives each year than cancer and chronic lower respiratory disease combined [1, 2]. The vast majority of CVD events are preventable, but the burden of CVD has been growing faster than the ability to tackle it. Identifying novel targets for reducing CVD risk is a critical step toward reversing current CVD incidence trends.

Psychosocial factors have been increasingly recognized as risk factors for CVD [3]. Indeed, depression and anxiety symptoms have been found to predict cardiovascular conditions with similar effect sizes as more traditional risk factors like smoking and obesity [4]. Over the past few decades, research has documented links between experiences of trauma with CVD onset [5, 6]. Trauma (e.g., natural disasters, unwanted sexual contact) is highly prevalent; the majority of individuals will experience a psychological trauma in their lifetime [7, 8]. Lifetime trauma exposure has been linked to a range of cardiovascular outcomes across studies, including coronary heart disease (CHD), myocardial infarction, and stroke, even when accounting for potential confounders and pathway variables [5, 9–12] Furthermore, in recent years, the American Heart Association has drawn attention to these issues in Scientific Statements highlighting the literature linking childhood adversity [13] and traumatic stress [14] with cardiovascular risk.

Given evidence of associations between trauma with CVD, there have been numerous efforts to understand underlying mechanisms. Identifying pathways linking trauma with CVD can inform targeted screening and intervention efforts to offset elevated cardiovascular risk. In this narrative review, we summarize the evidence for psychological and biological mechanisms linking trauma with CVD risk. Although not a systematic review, we present a comprehensive examination of the empirical literature on mechanisms, focusing in particular on mechanisms that have not received as much attention in the literature. Further, despite a robust preclinical literature, we highlight research in humans in this review. To inform future research in this area, we also describe various methodologies for measuring mechanisms, especially potential biological pathways. Finally, we highlight remaining gaps in understanding and recommend future directions to advance research in this area.

## Psychological mechanisms linking trauma with CVD risk

Adverse psychological responses to traumatic events have been posited as a key mechanism linking trauma with poor physical health [6]. Although many individuals are resilient after trauma, a sizeable proportion subsequently experience emotional difficulties [15]. Contingent upon trauma exposure, posttraumatic stress disorder (PTSD) is the quintessential trauma-related mental disorder [16]. However, PTSD is not the only mental disorder that can onset after trauma; other stress-related conditions (e.g., acute stress disorder) and mood, anxiety, and substance use disorders can all develop in response to a traumatic event [17–21]. Furthermore, PTSD is often comorbid with depression, anxiety, and substance misuse. Here, we summarize and evaluate the empirical evidence for manifestations of posttraumatic psychopathology as psychological mechanisms linking trauma with CVD risk.

### PTSD

The vast majority of research on posttraumatic psychopathology and CVD has focused on PTSD, and PTSD has been increasingly recognized as a key psychological mechanism underlying elevated cardiovascular risk after trauma [22]. In an initial meta-analysis of six studies, PTSD was associated with a 55% higher rate of incident CHD; although attenuated when adjusting for depression, the pooled hazard ratio (HR) for the PTSD-CHD association still provided evidence of an effect (HR = 1.27, 95% CI: 1.08–1.49) [23]. A more recent meta-analysis of nine longitudinal studies estimated that PTSD was associated with a 61% higher rate of incident CHD; again, depression did not account for the PTSD-incident CHD relation entirely (HR = 1.46, 95% CI: 1.26–1.69) [24]. In addition to this meta-analytic evidence, methodologically rigorous, longitudinal research has demonstrated that PTSD precedes and predicts a range of cardiovascular conditions, including myocardial infarction, stroke, venous thromboembolism, heart failure, and atrial fibrillation [12, 25–36]. Furthermore, PTSD has been associated with numerous cardiometabolic risk factors (e.g., hyperlipidemia, hypertension, diabetes, obesity) [37–40]. In these studies, PTSD has been measured in various ways, including symptom questionnaires [12, 29], clinical interview-based diagnoses [27], and diagnostic codes in electronic health records [25, 35].

For years, PTSD and CVD was studied in predominantly male veteran samples. This limited the ability to draw conclusions about civilians and women—notable shortcomings given the wide-ranging nature of trauma and established sex differences in PTSD and CVD [7, 41]. However, prospective research in population-based health registry samples has addressed this gap [25, 26]. In addition, longitudinal research has demonstrated associations between PTSD and incident CVD in community-dwelling women and women veterans, with effect sizes similar to those observed in men [12, 28, 35].

Together, this work provides substantial epidemiologic support that PTSD may increase risk of CVD. Indeed, in light of this evidence—and the potential public health implications—the National Heart, Lung, and Blood Institute held a workshop in 2018 to outline important directions for future research [42]. One such direction was to extend the research in observational cohorts and improve causal inference with Mendelian randomization (MR) using results from large-scale genome-wide association studies (GWAS) of PTSD and CVD. MR is an instrumental variable method that uses genetic instruments (which can be derived from publicly available GWAS summary statistics) to address the causal relation between a risk factor and health outcome [43]. Two MR studies on PTSD and cardiovascular risk have been conducted, and they suggest that genetically determined PTSD predicts hypertension [44] and CHD [45]. In contrast, no evidence for genetic predisposition for hypertension predicting PTSD was observed [44], and genetic predisposition for CHD was associated with *reduced* PTSD symptom severity [45]. The latter finding is contrary to evidence from cohort studies suggesting that cardiovascular events can serve as an index trauma for PTSD [22].

### Other posttraumatic psychopathology

Even though posttraumatic psychopathology can manifest in various ways, research that comprehensively considers a constellation of posttraumatic psychopathology as predictors of CVD is lacking. Research in national health registry samples has considered other stress-related disorders that—like PTSD—are contingent upon experiencing a severely stressful event (e.g., acute stress reaction, adjustment disorder); these stress-related mental disorders were associated with elevated risk of incident cardiovascular events and conditions [25, 26]. However, research in trauma-exposed samples has generally treated other mental disorders (e.g., depression) as confounders when evaluating PTSD and CVD, rather than investigating these other expressions of posttraumatic psychopathology as CVD predictors themselves [12, 28, 29]. This gap in the literature is notable, because depression, anxiety, and substance misuse are associated with CVD in non-trauma-exposed samples [46–48].

The predominant focus on PTSD has thus resulted in an incomplete characterization of the psychopathological effects of trauma exposure on subsequent CVD risk. Moreover, initial evidence suggests that considering psychiatric comorbidities after trauma may help identify those most at-risk for adverse physical health outcomes. Compared to women without trauma or depression, trauma-exposed women with high PTSD and depressive symptom levels had a nearly four-fold greater risk of all-cause mortality, plus higher rates of death from CVD [49]. These findings highlight the importance of considering co-occurrence of mental disorders when studying cardiovascular risk after trauma.

### Downstream pathways and shared genetic risk

Numerous behavioral and biological changes associated with posttraumatic psychopathology may subsequently contribute to cardiovascular risk. Here, we highlight some of these pathways, often focusing on ones associated with PTSD in particular as it has received the most attention within the literature. We describe additional biological mechanisms relevant to trauma and related psychopathology in more detail in Section 3.0. We also describe evidence for shared genetic factors that may increase risk of psychopathology and CVD after trauma.

Meta-analytic evidence has linked PTSD to numerous poor health behaviors and conditions, including physical inactivity, unhealthy diet, smoking, and obesity [50]. Additionally, medication nonadherence and greater substance use and abuse have been observed in individuals with PTSD [51, 52]. Other psychopathology such as depression and anxiety have also been linked to unhealthy behaviors [53–55]. Although numerous studies detect an association between PTSD and CVD even when accounting for these behaviors, effect sizes are often attenuated, suggesting these factors explain some of the excess cardiovascular risk in individuals with posttraumatic psychopathology [12, 27–29]. For example, unhealthy behaviors and obesity may contribute to metabolic dysregulation and related conditions (e.g., insulin resistance, metabolic syndrome, diabetes) that, in turn, increase vulnerability to CVD [56]. Furthermore, some research suggests that PTSD may contribute to psychopathology like depression, which—in turn—may contribute to unhealthy behaviors like physical inactivity and smoking, thereby increasing CVD risk [57]. This research demonstrates that considering various psychological consequences of trauma may help elucidate drivers of CVD-relevant pathophysiology.

A substantial literature has also described a strong connection between PTSD and dysregulation of the biological stress response, including the hypothalamic-pituitary-adrenal (HPA) axis and sympathetic-adrenal-medullary (SAM) system; changes in these systems may produce a cascade of effects conducive to poor cardiovascular health. Studies of individuals with PTSD have reported decreased cortisol levels, increased sensitivity of glucocorticoid receptors, and enhanced negative feedback of the HPA axis, although inconsistencies in findings across studies exist and may reflect differences in trauma exposure, manifestations and/or duration of posttraumatic psychopathology, and methodological factors [58–60]. In addition, PTSD has been characterized by hyperarousal of the sympathetic nervous system (SNS; e.g., elevated heart rate, blood pressure, skin conductance) and diminished parasympathetic activity (e.g., lower heart rate variability reflecting reduced vagal tone) [59–61]. Changes in HPA and autonomic functioning have implications for cardiovascular health (e.g., excessive catecholamines from SNS hyperreactivity can induce cardiac injury [62]), and they can influence other cardio-relevant biological mechanisms described in Section 3.0 (e.g., inflammation, oxidative stress) [63]. Other psychopathology such as depression has been characterized by HPA axis and autonomic dysfunction as well, although often in distinct ways from PTSD (e.g., depression is characterized by reduced feedback inhibition of the HPA axis, whereas PTSD is characterized by enhanced negative feedback) [58, 64], again suggesting a need to examine comprehensively the psychological sequelae of trauma when delineating these pathways.

Finally, shared genetic risk factors may also contribute to poor mental and cardiovascular health after trauma. For example, one study found associations between candidate genes for PTSD and CVD; of the 87 PTSD candidate risk genes, 37 were also risk genes for CVD, with many implicated in pathways related to immune function [65]. Research using results from GWAS of PTSD, depression, and cardiovascular outcomes has also pointed to positive genetic correlations between these mental and cardiovascular conditions [44, 66]. These results thus provide initial evidence for genetic overlap between PTSD and depression with cardiovascular risk, situating these conditions in a shared genetic milieu.

## Biological mechanisms linking trauma with CVD risk

**Fig. 1:**
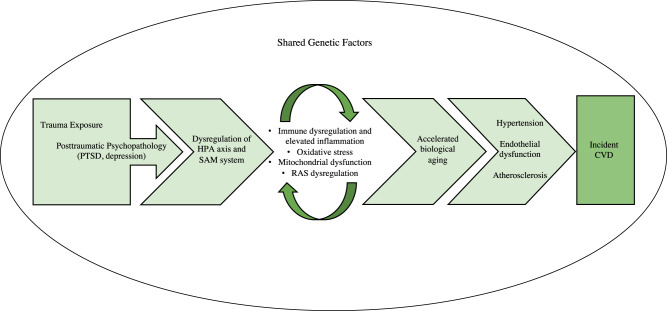
Conceptual model of key psychological and biological mechanisms linking trauma exposure with incident cardiovascular disease (CVD). Experiences of trauma and severe stress precede manifestations of posttraumatic psychopathology, such as posttraumatic stress disorder (PTSD) and depression. Subsequent dysregulation of biological stress response systems, including the hypothalamic-pituitary-adrenal (HPA) axis and sympathetic-adrenal-medullary (SAM) system, can contribute to further dysregulation in several interconnected biological systems, potentially leading to immune dysregulation and elevated inflammation, oxidative stress, mitochondrial dysfunction, and dysregulation of the renin-angiotensin system (RAS). Not only can these biological processes further influence one another (as indicated by the recursive arrows), but they can also contribute to accelerated biological aging. Together, these biological alterations can lead to the accumulation of intermediary cardiovascular risk factors, such as hypertension, endothelial dysfunction, and atherosclerosis, which—in turn—increase risk of developing CVD. Furthermore, these psychological and biological processes may unfold after trauma within a milieu of shared genetic risk.

Both trauma and posttraumatic psychopathology can contribute to downstream processes that increase risk of CVD. For example, as described in Section 2.3, PTSD is characterized by dysregulation of the HPA axis and SAM system [58–60], and trauma exposure—even without trauma-related psychopathology—is associated with similar dysfunction, although generally to a lesser degree than PTSD [67, 68]. These physiological changes can contribute to alterations in biological processes relevant to cardiovascular health, including immune dysregulation and elevated inflammation, oxidative stress, mitochondrial dysfunction, dysregulation of the renin-angiotensin system, and accelerated biological aging (Fig. 1). For each of these processes, we briefly introduce the underlying biology, describe links to CVD and other biological mechanisms, and review different measurement approaches to support the incorporation of these mechanisms in future studies. We then summarize and evaluate the empirical evidence for these biological mechanisms, first focusing on links with trauma and then with PTSD. Although some of these mechanisms are related to other psychopathology (e.g., depression [69, 70]), we focus on PTSD given the predominance of this disorder in the traumatic stress-CVD literature to date. Additionally, we provide details for some exemplar studies for each biological mechanism to convey further information about notable methods and key findings (Table 1).

**Table 1:** Detailed entries for example studies of biological mechanisms potentially linking trauma and/or PTSD with cardiovascular disease risk.

Study	Sample	Design	Measure(s) of trauma and/or PTSD	Measure(s) of biological mechanism	Key comparison	Summary of key findings	Notable points
**Immune dysregulation and elevated inflammation**							
Tawakol et al. [76]	13 adults with a history of PTSD	Cross-sectional	Participants had a history of PTSD based on the SCID-IV and CAPS; perceived stress assessed with the PSS-10	Imaging-based marker of arterial inflammation capturing cellular glycolysis (^18^F-FDG-PET); CRP levels	Association between perceived stress and arterial and peripheral inflammation among individuals with a history of PTSD	• Perceived stress was positively correlated with amygdalar activity, arterial inflammation, and CRP levels • Amygdalar activity mediated the majority of the association between perceived stress and arterial inflammation	• This study utilized a unique, imaging-based measure of arterial inflammation • Example of integrating multiple measures of inflammation
Carvalho et al. [90]	Genetic data from the PGC-PTSD Working Group (23,185 PTSD cases and 151,309 trauma-exposed controls) and the CHARGE Inflammation Working Group (148,164 individuals)	Cross-sectional	Genetically determined PTSD based on genome-wide summary statistics from the PGC-PTSD	Genetically determined CRP based on genome-wide summary statistics from the CHARGE Inflammation Working Group	Bidirectional associations between genetically predicted CRP and PTSD	• In Mendelian randomization analyses, genetically predicted PTSD and CRP showed significant bidirectional positive associations with one another	• This study utilized Mendelian randomization methods, which can help to illuminate causal relations using genetic data
**Oxidative stress**							
Atli et al. [110]	102 adults	Cross-sectional	PTSD diagnosis determined using the SCID-IV; PTSD symptom severity assessed with the PCL-C	Serum markers of antioxidant enzyme activity (PON1) and lipid peroxidation (MDA)	Compared PON1 activity and MDA levels across 3 groups (earthquake survivors with PTSD, earthquake survivors without PTSD, and healthy controls without a history of trauma)	• The PTSD group showed significantly elevated MDA levels and decreased PON1 activity compared to healthy controls • PTSD symptom severity was positively correlated with MDA levels • No significant differences between earthquake survivors with and without PTSD	• This study assessed oxidative stress with markers that reflect potential damage to due to oxidants and antioxidant enzyme activity • Comparing trauma-exposed individuals with and without PTSD to a no trauma control group provided an opportunity to assess differences due to trauma vs. trauma-related psychopathology
Boeck et al. [107]	30 adult postpartum women with and without a history of childhood maltreatment	Cross-sectional	Childhood maltreatment assessed with the CTQ	Serum metabolite markers of oxidative stress (arginine:citrulline ratio) and antioxidant capacity (L-carnitine and acetylcarnitine)	Association between childhood maltreatment and arginine:citrulline ratio, free L-carnitine, and acetylcarnitine	• Greater severity of childhood maltreatment was associated with higher levels of oxidative stress (greater arginine:citrulline ratio and lower L-carnitine and acetylcarnitine levels)	• Example of assessing serum metabolite markers reflecting greater oxidative stress and reduced antioxidant activity
**Mitochondrial dysfunction**							
Brunst et al. [148]	147 adult prenatal/ postpartum women enrolled in the PRISM Study	Longitudinal (PTSD symptoms assessed prenatally, mtDNAcn assessed postpartum)	Prenatal PTSD symptoms assessed with the PCL-C	Placental mtDNAcn	Association between prenatal PTSD symptoms and placental mtDNAcn	• Greater prenatal PTSD symptom severity was associated with lower placental mtDNAcn • Non-White individuals had higher PTSD symptom levels and lower mtDNAcn than White individuals; some differences in the PTSD-mtDNAcn relation emerged by race/ethnicity	• Longitudinal design in a prospective pregnancy cohort is a strength • Considered potential racial/ethnic differences in the relations between maternal psychological experiences and a biological marker relevant to maternal-fetal health
Gumpp et al. [137]	102 mother- newborn dyads	Cross-sectional	Childhood maltreatment assessed with the CTQ	Measures of mitochondrial respiration (e.g., routine respiration, ATP-turnover- related respiration) and intracellular mitochondrial density (citrate synthase activity) in UBMCs of newborns and in PBMCs of mothers	Compared measures of mitochondrial respiration and density in mothers with and without childhood maltreatment and in their newborns	• Childhood maltreatment was significantly positively associated with some measures of mitochondrial respiration and with citrate synthase activity in mothers • Maternal history of childhood maltreatment had a small but nonsignificant positive effect on citrate synthase activity in newborns	• Considered multiple measures of mitochondrial function and content • Measured mitochondrial dysfunction in mothers and newborns, though evidence for maternal childhood maltreatment alterations in mitochondrial measures was lacking
**Renin-angiotensin system dysregulation**							
Terock et al. [161]	3,092 adults enrolled in the SHIP	Cross-sectional	PTSD diagnosis based on the SCID-IV	Plasma concentrations of renin and aldosterone	Compared renin and aldosterone levels, and aldosterone:renin ratio, across 3 groups (individuals with PTSD, trauma-exposed individuals without PTSD, and individuals without a history of trauma)	• Relative to those with no trauma history, trauma-exposed individuals with and without PTSD showed elevated renin levels, with effects most pronounced for individuals with PTSD • Greater trauma load was positively associated with renin level and negatively associated with aldosterone:renin ratio • PTSD continued to significantly predict renin when adjusting for trauma load	• Comparing trauma-exposed individuals with and without PTSD to a no trauma control group provided an opportunity to assess differences due to trauma vs. trauma-related psychopathology
Seligowski et al. [164]	840 trauma-exposed adults (Primary sample); 116,389 adults in the Partners Healthcare Biobank (Replication sample)	Cross-sectional	PTSD symptoms assessed by the M-PSS (Primary sample); PTSD diagnosis based on diagnostic codes in the electronic health record (Replication sample)	Treatment with ACE-Is or ARBs (RAS blocker)	Association between ACE-I/ARB status and PTSD, plus differences by sex and race	• In the Primary sample, among individuals treated with ACE-Is/ARBs, women had higher PTSD symptom levels than men • In the Replication sample, ACE-I/ARB treatment was significantly associated with a lower rate of PTSD diagnosis • The relation between ACE-I/ARB treatment and PTSD diagnosis was significant among White but not Black individuals in the Replication sample	• Targeted medication use provides a unique measure of RAS functioning • Large sample for replication derived from a biorepository database • Considered sex and race as potential moderating factors
**Accelerated biological aging**							
Copeland et al. [220]	381 individuals in the Great Smoky Mountains Study followed from childhood to young adulthood	Longitudinal (Early life adversity assessed in childhood; DNAm measured in childhood and adulthood)	Dimensions of early life adversity (i.e., threat, material deprivation, loss, and unpredictability) assessed with the CAPA	DNAm from bloodspots; DNAm age estimated using elastic nets to predict chronological age; change over time=difference score between childhood and adulthood DNAm age estimates	Associations between early life adversity (dimensions and cumulative measure) with DNAm age, cross-sectionally in childhood and in terms of change over time	• No significant early life adversity- DNAm age associations in childhood in cross-sectional analyses • Greater cumulative early life adversity was significantly associated with increased DNAm aging from childhood to adulthood • No unique effects emerged for adversity dimensions when testing all dimensions simultaneously	• DNAm age was assessed longitudinally in childhood and adulthood • Example of studying longitudinal within-in person changes in DNAm age rather than examining between-group differences at a given time point
Mehta et al. [226]	40 adult paramedicine students	Longitudinal (PTSD and DNAm measured before and after trauma exposure)	PTSD symptom severity assessed with the PCL-5	DNAm from saliva; DNAm age calculated using Horvath, GrimAge, and Skin & Blood Age clocks; Horvath age was residualized on chronological age and GrimAge and Skin & Blood Age were chronological age-adjusted	Associations between PTSD symptom severity and DNAm age, cross-sectionally and longitudinally	• Significant positive associations cross-sectionally and longitudinally were observed for PTSD symptom severity and epigenetic age acceleration for the Horvath and GrimAge clocks • No significant associations observed between PTSD symptoms and Skin & Blood age acceleration	• Longitudinal design in which DNAm was measured before and after trauma exposure is a strength

^*18*^*F-FDG-PET*^18^F-fluorodeoxyglucose positron emission tomography, *ACE-I* angiotensin-converting enzyme inhibitor, *ARB* angiotensin receptor blocker, *CAPA* Child and Adolescent Psychiatric Assessment, *CAPS* Clinician-Administered PTSD Scale, *CHARGE* Cohorts for Heart and Aging Research in Genomic Epidemiology, *CRP* C-reactive protein, *CTQ* Childhood Trauma Questionnaire, *DNAm* DNA methylation, *MDA* malondialdehyde, *M-PSS* Modified PTSD Symptom Scale, *mtDNAcn* mitochondrial DNA copy number, *PBMCs* peripheral blood mononuclear cells, *PCL-5* PTSD Checklist for DSM-5, *PGC-PTSD* Psychiatric Genomics Consortium-PTSD, *PON1* paraoxonase 1, *PRISM* Programming of Intergenerational Stress Mechanisms Study, *PSS-10* Perceived Stress Scale, *PTSD* posttraumatic stress disorder, *RAS* renin-angiotensin system, *SCID-IV* Structured Clinical Interview for DSM-IV, *SHIP* Study of Health in Pomerania, *UBMCs* umbilical cord blood mononuclear cells.

### Immune dysregulation and elevated inflammation

The immune system encompasses cells, chemicals, and processes that defend the body from noxious stimuli, and inflammation is a key component of the immune response [71]. Inflammatory responses involve a complex cascade of signaling molecules; although acute increases in inflammation in response to injury or infection are critical for health, a chronic state of inflammation can contribute to disease. Inflammation has been implicated in cardiovascular event onset, disease progression, and adverse prognosis [72]. For example, epidemiologic studies have demonstrated that elevated inflammatory biomarkers predict CVD [73], and atherosclerosis—the narrowing of arteries due to plaque accumulation, a major CVD risk factor—is now conceptualized as an inflammatory condition [72].

Immune- and inflammation-related processes can be measured in research in various ways. Quantifying levels of peripheral inflammation-related biomarkers, such as C-reactive protein (CRP) and interleukin-6 (IL-6), are common in the field of traumatic stress [74, 75], although imaging-based approaches that capture cellular glycolysis (e.g., fluorodeoxyglucose positron emission tomography) have begun to be used to detect vascular inflammation [76, 77]. Immune function can also be captured, for example, by considering in vitro stimulated measures of inflammatory cytokine- and/or chemokine-producing immune cells [78].

Trauma, PTSD, and inflammation-related biomarkers have been studied extensively, with several meta-analyses on these relations. A meta-analysis of 36 independent samples found moderate positive correlations between trauma exposure and several inflammation-related biomarkers [CRP, IL-1β, IL-6, tumor necrosis factor-α (TNF-α)]; no associations were observed for fibrinogen, IL-2, IL-4, IL-8, or IL-10 [75]. In addition, childhood trauma was associated with elevated CRP, IL-6, and TNF-α levels with small effects in a meta-analysis of 25 studies [79]. Several systematic reviews and meta-analyses examining PTSD and inflammatory markers have also been conducted [74, 80–82]. In the most recent meta-analysis of 54 studies examining over 15 inflammatory markers, elevated levels of CRP (moderate effect), IL-6 (large effect), and TNF-α (large effect) were observed in individuals with PTSD compared to controls; there was also weak evidence of a small effect for PTSD and IL-1β [82]. Together, these results suggest that trauma and PTSD are associated with elevations in peripheral inflammatory markers. However, most studies have been cross-sectional, which limits the ability to draw directional and causal conclusions. Indeed, bidirectional associations between PTSD and inflammation have been postulated, and the relatively few existing longitudinal studies have yet to provide robust evidence that a proinflammatory milieu leads to PTSD and/or that trauma and PTSD promote inflammation [83]. In addition to studies of peripheral biomarkers, preliminary research in small samples of individuals with PTSD has examined vascular inflammation using imaging-based approaches, although findings have been mixed [76, 77].

Nevertheless, support for links between trauma, PTSD, and inflammation has been observed across omics studies. For example, hypothesis-free genetic, epigenetic, and gene expression studies of PTSD have identified genes related to the immune system [e.g., genes in the HLA region and those encoding inflammatory cytokines (IL-8, IL-16)] [84–86], which parallels work with candidate genes [65]. Additionally, some studies have found that variation in and/or methylation of immune-relevant genes underlie associations of PTSD and inflammation. For instance, in some (but not all) studies of military veterans, methylation of the Absent in Melanoma 2 (*AIM2*) gene, which has been implicated in the inflammatory response, partially accounted for associations of PTSD with elevated inflammatory markers [87–89]. Furthermore, MR research has begun to address causality in the PTSD-inflammation and inflammation-PTSD relations, with an initial study documenting small bidirectional associations between PTSD and CRP [90]. Finally, several metabolomics studies have identified metabolites related to inflammation and immune function (e.g., sphingolipids, glycerophospholipids) in profiles distinguishing individuals with and without PTSD [91–93].

### Oxidative stress

Oxidative stress may also underlie associations of trauma and PTSD with CVD. Cellular energy production generates pro-oxidants [e.g., reactive oxygen species (ROS)] as naturally occurring byproducts, and regulation by an antioxidant defense system is key for cellular functioning [94, 95]. Oxidative stress arises when there is an imbalance between ROS and other pro-oxidant molecules and their neutralization by antioxidants [94, 95]. A prolonged state of oxidative stress can result in cellular damage and death. Oxidative stress and inflammation often co-occur and influence one another; inflammatory signaling can trigger ROS production, and oxidative stress stimulates the immune response [94, 95]. Furthermore, oxidative stress has been linked to CVD [96]. Traditional cardiovascular risk factors like hyperlipidemia and hypertension contribute to ROS production [97], and oxidative stress can contribute to endothelial dysfunction [98]—an early indicator of reduced capacity of blood vessels to respond to cardiovascular demand [99]—and atherosclerosis [97].

Although it is challenging to measure pro-oxidant molecules like ROS directly due to short half-lives and low concentrations, approaches have been developed to quantify oxidative stress [100]. For example, biomarkers indicative of damage induced by oxidative stress to lipids, proteins, and DNA (e.g., F_2_-isoprostanes, protein carbonyls, 8-hydroxy-deoxyguanosine, respectively) can be measured using immunoassays or mass spectrometry [100, 101]. Alternatively, antioxidant-based measures can be used to capture oxidative stress. Such approaches include quantifying antioxidant enzyme levels and activity, as well as the antioxidant capacity in bodily fluids—an in vitro metric that uses color or fluorescence changes to quantify the extent to which pro-oxidants are counteracted by antioxidants [101, 102].

Although some research has linked life stress (e.g., chronic caregiving, examination stress) with greater oxidative stress [103, 104], few studies have considered traumatic experiences, with all focused on early-life adversity. In two small studies of adolescents, those with a history of early-life adversity had elevated markers of oxidative damage to lipids (F_2_-isoprostanes) and proteins (protein carbonyls), and an increased enzymatic pro-oxidant/antioxidant defense ratio and lower non-enzymatic antioxidant capacity [105, 106]. Furthermore, one small study of postpartum women found that more severe childhood maltreatment was associated with serum metabolites associated with greater oxidative stress and lower antioxidant capacity [107]. However, some studies in children [108] and adults [109] have failed to detect associations between early-life adversity and oxidative stress markers. Notably, all studies employed retrospective reports of early-life adversity in relatively small samples, and research on lifetime experiences of trauma is lacking.

Whereas the research on trauma and oxidative stress has been limited, numerous studies have assessed oxidative stress in individuals with PTSD. A recent meta-analysis did not find an association between PTSD with malondialdehyde (a product of lipid peroxidation) or two antioxidant enzymes (catalase, paraoxonase-1) [82], although only five studies were included and three drew participants from the same sample [110–114]. Overall, additional studies of PTSD and oxidative stress—most conducted among combat veterans [115–118]—have had mixed results [119–121]. Furthermore, studies have varied widely in measures of oxidative stress, which complicates comparisons. Some additional evidence for a link between PTSD and oxidative stress comes from omics studies. For example, a variant in the retinoic acid orphan receptor (*RORA*) gene, involved in oxidative stress-related biology, was identified in the first GWAS of PTSD [122], and findings from epigenetic and gene expression studies of PTSD have implicated genes related to oxidative stress [123–125]. Additionally, metabolite profiling studies comparing individuals with and without PTSD have identified differences in metabolites related to oxidative stress (e.g., proline, hydroxyproline, 4Z,15E-bilirubin IXa) [91, 93, 126].

### Mitochondrial dysfunction

Given their critical role in energy production, mitochondria are key producers of ROS that are closely connected with immune modulation and inflammation-related processes [127]. Mitochondria have their own DNA—mitochondrial DNA (mtDNA)—with critical genes for energy production. In addition, mitochondria can change in structure and function as a result of environmental signals they detect; some of these changes can contribute to mtDNA damage, which can lead to mitochondrial dysfunction and adversely impact energy metabolism, ROS production, and signaling [127, 128]. Changes in mitochondrial structure and function can contribute to cardiometabolic dysregulation, including obesity, hypertension, and hyperlipidemia [129], and mitochondrial dysfunction can lead to endothelial dysfunction and atherosclerosis [130].

Multiple mitochondria-relevant metrics exist; measuring aspects of mitochondria function and content provides the most comprehensive assessment [127]. Functional measures include quantifying oxygen consumption rate in living cells and respiratory chain enzymatic activity in frozen cells [131]. Additionally, mitochondria morphology can be assessed with electron microscopy [132], and activity of the mitochondrial enzyme citrate synthase can be quantified to measure the density of the mitochondrial network per cell [133–135]. Several mtDNA-related measures [e.g., mtDNA copy number (mtDNAcn) per cell, mutations, deletions] can also be assessed as potential indicators of mitochondrial dysfunction [127, 136]. Additionally, circulating cell-free mtDNA (ccf-mtDNA) is a pro-inflammatory mitochondria-derived signaling molecule detectable in peripheral samples [127].

As with research on oxidative stress, most research has examined stress and mitochondrial dysfunction [127, 131, 133], but some studies have examined early-life adversity. One small sample of postpartum women found that more severe experiences of childhood maltreatment were associated with greater mitochondrial routine physiological activity and augmented energy and ROS production, but not with citrate synthase activity [107]. These findings were partially replicated in a larger sample of mother-newborn dyads in the My Childhood-Your Childhood study from the same research group; mothers with vs. without a history of childhood maltreatment had higher mitochondrial routine physiological activity, augmented energy production, and higher citrate synthase activity [137]. However, no associations between maternal childhood maltreatment and newborns’ mitochondrial measures were observed. My Childhood-Your Childhood study investigators also examined whether maternal childhood maltreatment was related to differential change in mitochondrial metrics within the first year after birth [138]. At the 1-year follow-up, childhood maltreatment-related differences in mitochondrial function and intracellular density at baseline (reported in Gumpp et al. [137]) were no longer observed, suggesting that these mitochondrial changes may have only been detectable after the physiological demands of childbirth.

Whereas links between early-life adversity and mitochondrial function have been examined predominantly in postpartum women, associations between early-life adversity and mtDNAcn have been investigated in more diverse samples [136]. For example, history of childhood maltreatment was associated with greater mtDNAcn in adults [139] and children [140], however, in one additional study, the positive association between childhood sexual abuse and mtDNAcn was only observed in individuals with depression [141]. In contrast, maternal lifetime trauma exposure was negatively associated with mtDNAcn in placenta, but not cord blood [142]. Additionally, no significant differences in mtDNAcn were observed in Holocaust survivors or their descendants compared to age-matched controls [143]. With respect to circulating mitochondrial-related markers, two studies of traumatic injury patients observed higher concentrations of mtDNA in plasma compared to controls [144, 145], and women who experienced sexual trauma during adolescence had significantly more ccf-mtDNA than women with no sexual trauma or who experienced sexual trauma during childhood or adulthood [146].

Studies of PTSD and mitochondria are even fewer than those investigating trauma and mitochondrial dysfunction. In male combat veterans, individuals with vs. without a current PTSD diagnosis had lower mtDNAcn; this was driven by individuals with mild or severe symptoms [147]. In a prospective pregnancy cohort, greater prenatal PTSD symptoms were associated with lower mtDNAcn in placenta [148]. However, in trauma-exposed women, there was no significant correlation between current PTSD symptoms and ccf-mtDNA [146]. Indirect support for a link between PTSD and mitochondrial function also comes from mitochondrial GWAS [149], along with epigenetic [150], gene expression [125], and metabolomics studies [93].

### Renin-angiotensin system (RAS) dysregulation

Dysregulation of the RAS, which interacts with the HPA axis and SAM system, represents another biological mechanism potentially linking traumatic stress with CVD risk [151–153]. The RAS is a key regulator of blood pressure, along with fluid and salt balance [152, 154]. The enzyme renin leads to the production of angiotensin I and II in blood and tissues. As the main effector molecule of the RAS, angiotensin II has numerous functions, including constricting blood vessels, stimulating sodium reabsorption and triggering the adrenal cortex to release aldosterone, and promoting norepinephrine release [152]. RAS activation can contribute to cardiovascular risk via elevated SNS activity, blood pressure, inflammation, oxidative stress, and endothelial dysfunction [63, 154], and elevated renin predicts myocardial infarction [155, 156]. Furthermore, elements of the RAS have become cardiovascular pharmacotherapy targets; angiotensin converting enzyme inhibitors (ACE-Is) and angiotensin receptor blockers (ARBs) are RAS blockers commonly used to manage hypertension and have been shown to reduce SNS activity [63, 154].

RAS components can be assessed from peripheral samples. For example, plasma renin activity, a measure of renin’s capacity to generate angiotensin I, can be determined by activity assay; immunoassays can also be used to estimate renin concentration in plasma [157, 158]. Additionally, immunoassays can be used to quantify endogenous angiotensins (e.g., angiotensin II) and the steroid hormone aldosterone [158, 159].

Although acute stress has been associated experimentally with increases in RAS components (renin, aldosterone) [153], only three studies have examined trauma, PTSD, and the RAS. In a large general population sample from the Study of Health in Pomerania, greater childhood trauma was associated with higher plasma concentrations of aldosterone—but not renin—and greater adulthood trauma exposure was associated with higher plasma renin—but not aldosterone—concentrations [160]. In a second Study of Health in Pomerania investigation, individuals with trauma but without PTSD and individuals with PTSD had elevated renin (but not aldosterone) levels compared to individuals without trauma; those with PTSD showed the most pronounced renin elevations [161]. Additionally, middle-aged women with chronic PTSD had lower aldosterone levels compared to women without trauma [162]. Although findings have been mixed from the few studies examining traumatic stress and RAS components, further indirect evidence for a PTSD-RAS link comes from ACE-I/ARB studies. Specifically, use of ACE-Is/ARBs (but not other antihypertensive medications) has been associated with lower PTSD symptoms [163, 164], with some evidence of moderation by genetic variation or sex [164, 165]. However, there was no evidence for clinical benefit of ARB use for PTSD in a 10-week randomized, placebo-controlled trial [166].

### Accelerated biological aging

Many of the biological processes described above, including elevated inflammation and oxidative stress, may contribute to CVD via accelerated biological aging (BA). Indeed, trauma and PTSD have been linked to early-onset CVD [12, 34, 35], prompting interest in whether the pace of cellular aging may be hastened after trauma and contribute to premature disease [167]. Cellular markers of BA associated with aging-related process and CVD include telomeres, epigenetic clocks, and composite biomarker-based estimates. For example, telomeres—noncoding DNA sequences at the ends of chromosomes—shorten with age, although telomere attrition can accelerate due to environmental factors [168]. Cellular senescence is triggered when telomeres shorten to a particular length, and shortened telomeres predict mortality and incident CVD [169, 170]. Further, MR research suggests a causal link between telomere length and CVD [171]. Numerous “epigenetic clocks” have also been developed that estimate age based on DNA methylation (DNAm) data across the genome [172]. The extent to which these DNAm age estimates are accelerated relative to chronological age predicts mortality and CVD [173, 174]. In addition, BA estimators that integrate information from clinical biomarkers across multiple physiological systems have been developed that predict mortality and morbidity [175–177].

Multiple methods can be leveraged to quantify these BA measures. Telomere length can be measured in several specimens, including blood and saliva, using various methods (e.g., terminal restriction fragment length analysis by Southern blot, quantitative polymerase chain reaction with DNA analytes [178, 179]); however, tissue type and analytic approach have been found to influence reliability [179–181]. Recently, a DNAm estimator of telomere length was also developed [182]. Additionally, multiple epigenetic clocks are available, including first-generation clocks calibrated to predict chronological age (the pan-tissue Horvath clock [183] and blood-based Hannum clock [184]) and second-generation clocks trained to predict morbidity- and mortality-related outcomes (PhenoAge [176] and GrimAge [185]). Most of these clocks can be applied to DNAm from blood samples in adults, although the Horvath clock was calibrated across various tissues and in youths and adults [177, 183]. In addition, individual variability in the pace of biological aging was recently distilled into a single timepoint DNAm measure (DunedinPACE) [186]. Finally, composite biomarker estimates of BA can be calculated from various biomarkers (e.g., CRP, creatinine) often collected in clinical settings; several computational methods are available (e.g., Klemera-Doubal method, Phenotypic Age algorithm [175, 176]). Calculation of various DNAm age estimates and composite biomarker estimates is facilitated by the availability of online calculators and statistical packages [183, 187]. Interestingly, relatively low agreement has been found between various BA measures, suggesting that they capture different aspects of aging-related processes [188].

Trauma, PTSD, and telomeres have been studied extensively, as summarized in several systematic reviews and meta-analyses. Early-life adversity has generally been linked to shorter telomere length, despite heterogeneity across investigations [189–194]; in the largest meta-analysis of 41 studies in youths and adults, there was a small-to-medium overall association between early-life adversity and reduced telomere length [190]. Furthermore, there is some meta-analytic evidence that early adverse experiences characterized by threat—not deprivation or socioeconomic disadvantage—are associated with accelerated BA in youths (measured across telomere and DNAm age metrics) [195]. In several studies of early-life adversity and telomeres published since these review papers, most—but not all—have demonstrated negative associations between early-life adversity and telomere length [196–203], yet there has been mixed evidence for intergenerational transmission of trauma via telomere length [204–206]. Fewer studies have examined traumatic experiences in adulthood (e.g., military service, solitary confinement during war captivity, lifetime trauma) and telomere length, with mixed results [203, 207–209]. Research on PTSD and telomeres has also been summarized in reviews. One meta-analysis of five studies found a small overall effect of PTSD on shorter telomere length; as with research on early-life adversity, substantial heterogeneity was detected [191]. A more recent systematic review included 13 studies of PTSD and telomeres, with six finding a negative association, three (all in military samples) not detecting an association, one finding a positive association, and three finding mixed results [210]. More recent studies have continued to have inconsistencies or nuance in results, with some observing a negative PTSD-telomere length relation only for older individuals [211] or for re-experiencing symptoms [212], and others detecting a positive association between PTSD and telomere length [213, 214]. A few studies of telomeres in trauma-exposed individuals have also considered manifestations of posttraumatic psychopathology beyond PTSD, detecting shorter telomeres in former prisoners of war with greater depressive symptoms [215] and in women with comorbid PTSD and depressive symptoms [216].

Although fewer studies have examined associations between trauma, PTSD, and DNAm age compared to the telomere literature, this is an area of growing interest. A recent systematic review identified 10 studies of traumatic stress and DNAm age in adults; four of these examined early-life adversity, with one finding an association with advanced DNAm age relative to chronological age (i.e., epigenetic age acceleration) [217]. In a large meta-analysis of individuals across nine cohorts, childhood trauma was associated with Hannum (but not Horvath) epigenetic age acceleration, however only when measured with the Childhood Trauma Questionnaire [218]. Greater experiences of early-life adversity were also linked to DNAm age acceleration based on several epigenetic clocks in two large longitudinal cohorts [219, 220]. In youths, early-life adversity has been linked to epigenetic age acceleration, with nuances in the findings detected. For example, in the Avon Longitudinal Study of Parents and Children (ALSPAC) cohort, early-life adversity during early and middle childhood was associated with Hannum (but not Horvath) epigenetic age acceleration—suggesting sensitive periods, rather than cumulative or recency effects—when it comes to early-life adversity and BA [221]. Additional work in ALSPAC found that greater early-life adversity was associated with Horvath (but not Hannum) epigenetic age acceleration in adolescent girls and not boys [222]. In contrast, as noted in the Lim and colleagues [217] systematic review, relatively few studies have detected a link between lifetime trauma and epigenetic age acceleration, a finding echoed in more recent investigations [218, 223]. Research on PTSD and DNAm age has more consistently demonstrated evidence for epigenetic age acceleration, although there is variability in which epigenetic clocks have effects detected. The Lim and colleagues [217] systematic review and a recent review [224] reported that 5 of 7 studies and 7 of 11 studies, respectively, observed an association between PTSD and accelerated epigenetic age. Recent studies have a similar overall finding, although again which epigenetic clock is accelerated varies [225–228]. There is also initial evidence that other posttraumatic psychopathology may be relevant to DNAm age. For example, current alcohol use disorder—but not depression or generalized anxiety disorder—was associated with a faster pace of the Horvath epigenetic clock in a longitudinal study of veterans [229]. Additionally, although PTSD diagnosis was not related to pace of the epigenetic clock, avoidance and numbing symptoms of PTSD were predictors.

To date, only a handful of studies have investigated early-life adversity and composite biomarker-based BA measures. Across three studies in large longitudinal cohorts, early-life adversity was associated with more advanced BA based on the Klemera-Doubal method, Phenotypic Age algorithm, and a multi-biomarker indicator of pace of BA [230–232].

## Recommendations and future directions

There is now an extensive literature suggesting that CVD risk is elevated after trauma. The psychological response to trauma appears to be a key mechanism linking these experiences with adverse cardiovascular health. Not only is dysregulation of cardio-relevant biological processes generally more pronounced in those with posttraumatic psychopathology than with trauma alone [67, 68, 233], but studies that directly compare risk of incident CVD in trauma-exposed individuals with and without posttraumatic psychopathology generally detect larger effects in individuals with psychological distress after trauma [12, 34]. These findings have been observed in a variety of trauma-exposed samples using rigorous longitudinal designs that account for numerous potential confounders, and this work has been complemented by MR studies that further suggest a causal association. However, despite the many ways in which posttraumatic psychopathology manifests, the vast majority of research has focused on PTSD. This is a notable limitation, especially as the few studies considering multiple forms of posttraumatic psychopathology have often found more pronounced health risks in individuals with comorbid symptoms, suggesting the value of looking beyond just PTSD [49, 216].

Going forward, it is critical to consider whether other posttraumatic psychopathology has an etiologic effect on cardiovascular health. For example, it is unclear whether there are combinations and/or sequences of psychopathology after trauma that are particularly cardiotoxic, and whether these combinations vary for different groups (e.g., men vs. women), trauma types (e.g., interpersonal vs. non-interpersonal trauma), and cardiovascular outcomes. Furthermore, given racial and ethnic inequities in trauma exposure, risk of trauma-related psychopathology, access to treatment, and CVD risk, studies in diverse populations are critical [1, 234]. In addition, research is needed that extends beyond traditional diagnostic categories and investigates how transdiagnostic symptom dimensions relate to cardiovascular risk after trauma. Such an approach may identify key posttraumatic symptoms that may be targeted to reduce cardiovascular risk. For example, initial research suggests that fear-related symptoms after trauma may be particularly associated with cardiovascular risk [235–237].

Extensive research also suggests dysregulation of a variety of inter-related biological processes that may contribute to elevated cardiovascular risk both after trauma and in individuals with PTSD. Immune- and inflammation-related processes are some of the most well-studied and supported mechanisms to date, with evidence from meta-analyses and multiple omics studies. However, most investigations have been cross-sectional, and longitudinal research is needed to better understand risk processes after trauma. There is also considerable support for accelerated BA after trauma and in those with posttraumatic psychopathology, particularly for telomeres. Despite clear connections between stress-related biological systems that are dysregulated after trauma and in PTSD and the other biological mechanisms highlighted in this review (e.g., oxidative stress, mitochondrial dysfunction, RAS dysregulation), the latter processes have yet to be the subject of ample empirical study. Further, most studies have focused on early-life adversity—typically reported retrospectively—and sample sizes have generally been small and methods varied.

More comprehensive research on how experiences of trauma over the lifespan, and a range of posttraumatic psychopathology, relate to downstream biological processes is thus needed. Furthermore, although some research has considered potential sex differences in the traumatic stress-CVD relation [25, 26], research that extends this consideration to biological mechanisms is needed. For example, gonadal hormones (e.g., estradiol, testosterone) have documented effects on many of the biological mechanisms discussed in this review (e.g., inflammation, the RAS) [238, 239], and an important future direction is to consider sex differences in these mechanisms after trauma and potential consequences for CVD risk. Additionally, given the interconnections between processes, an integrative systems biology approach is likely to be valuable for understanding mechanisms contributing to cardiovascular risk. Preclinical experimental studies of trauma and PTSD offer elegant examples for studying processes across multiple levels of analysis and their impact on cardiovascular metrics [240, 241]. Although systems biology research is of growing interest in the traumatic stress field [242], this approach has yet to be implemented in trauma and CVD research. Additionally, examining dynamic changes in biological mechanisms in response to stress and trauma-related stimuli may shed light on how risk processes unfold—and how interventions may affect these processes [243, 244].

Ultimately, a more refined understanding of psychological and biological mechanisms can inform CVD intervention efforts. Initial evidence suggests that PTSD treatment may attenuate cardiovascular risk [245, 246], and a trial of gold-standard treatment for PTSD is underway to investigate potential impact on cardiovascular risk markers [247]. Although treating PTSD to improve mental health itself is an important goal, it is of interest to examine whether trauma-focused psychotherapies for PTSD may improve cardiovascular risk markers and related biological mechanisms directly or via reductions in posttraumatic psychopathology. Nevertheless, a longitudinal study in veterans did not find that clinically meaningful reductions in PTSD symptoms were associated with reduced incidence of CVD [248]. Investigating interventions that engage behavioral and/or biological mechanisms linking trauma with adverse cardiovascular health (e.g., anti-inflammatory or antioxidant treatments [95], physical activity interventions [249]) may also prove fruitful for developing multi-modal treatment approaches for reducing cardiovascular risk after trauma.
